# Regular caffeine consumption & subjective sleep quality: A systematic review

**DOI:** 10.1016/j.jarlif.2025.100005

**Published:** 2025-02-08

**Authors:** Duc Minh Phan, My Yen Lam, Minh Nguyet Trang

**Affiliations:** Faculty of Public Health, University of Medicine and Pharmacy at Ho Chi Minh city, 217 Hong Bang, Ward 11, District 5, Ho Chi Minh city, Vietnam

**Keywords:** Caffeine, Regular caffeine consumption, Sleep, Subjective sleep quality, Systematic review

## Abstract

Caffeine, the world's most consumed stimulant, is commonly used to combat fatigue, This review examined the unclear relationship between long-term caffeine consumption and subjective sleep quality, defined as satisfaction with sleep based on personal perception. Screening 6,908 studies, 10 were included. Results showed limited statistically significant associations, hindered by variability in study quality, data, and assessment methods. Influencing factors such as individual differences and study heterogeneity were identified. The review highlights the need for standardized tools and methodologies, urging future research to explore genetic, cultural, and timing factors to better understand caffeine's impact on sleep quality.

## Highlights

• First systematic review on regular caffeine consumption and subjective sleep quality addresses an important and widely relevant topic in today's society.
• Understanding the relationship between caffeine consumption and subjective sleep quality can help inform evidence-based guidelines and recommendations for both healthcare professionals and the general public.
• The systematic review synthesizes existing research on this topic, providing a comprehensive overview of the current state of knowledge and identifying gaps or inconsistencies in the literature.

## 1 Introduction

Caffeine is the most widely consumed food stimulant in the world [[1], [2], [3]]. Its ability to reduce drowsiness and fatigue makes it an integral part of daily life for many individuals seeking enhanced alertness and productivity [1]. Sleep problems are a growing public health issue, with 45 % of the population reportedly affected in 2016 [4]. Although many studies have explored this, the effect of regular caffeine consumption on perceived sleep quality is still unclear due to conflicting findings. Some evidence suggests that caffeine reduces total sleep time [5], while other findings indicate no significant effect [6]. Additionally, variations in study designs, caffeine measurement methods, and a lack of focus on subjective sleep quality complicate drawing definitive conclusions [7]. This gap in knowledge necessitates a comprehensive evaluation of the long-term effects of regular caffeine consumption on subjective sleep quality. Addressing this issue is essential for informing clinical recommendations, guiding lifestyle choices, and shaping public health policies aimed at improving sleep hygiene and overall well-being. This systematic review synthesizes evidence from previous studies to assess the association between regular caffeine consumption and subjective sleep quality. By addressing existing gaps, we aim to provide insights that are meaningful to individuals, clinicians, and policymakers, contributing to a more nuanced understanding of caffeine's role in sleep health.

## 2 Methods

### 2.1 Search strategy

The search strategy in the study was conducted on the Pubmed, Scopus, ScienceDirect databases from December 2022 to June 2023 to examine the association between regular caffeine consumption and subjective sleep quality. **Pubmed**: (“Caffeine ” OR “Coffee” OR “Tea”) AND (“Sleep”), filters: English, Results by year: 1931-2023; **Scopus**: (“Caffeine ” OR “Coffee” OR “Tea”) AND (“Sleep”), source type: Journal, Language: English, Document type: Article; **ScienceDirect**: (“Caffeine ” OR “Coffee” OR “Tea”) AND (“Sleep”), years: 1999-2023.

### 2.2 Sample selection criteria

Studies on the effects of caffeine consumption on sleep were primarily epidemiological studies and randomized controlled trials. ***Inclusion criteria:*** randomized controlled studies; epidemiological studies: cohort, case-control, cross-sectional studies; studies found would be selected according to the Prisma diagram; researches were not limited to subjects, age, race, sex, socioeconomic status, ethnicity and place of residence; research with no publication date limit. ***Exclusion criteria:* Read the title and summary*:*** systematic reviews; case reports; not related to the topic and research objectives; animal studies. ***Read the full article:*** results focused only on objective sleep quality or lacked a scoring scale; not full-text research article; duplicate articles; acute consumption (<1 month and not converted to average daily intake); not in English; units used for the amount of caffeine other than grams or milligrams; opinions, reviews, conference report. This study complied with the checklist for a systematic review following the Prisma procedure. As with other systematic reviews, evaluation of the quality score of the studies were an important condition. We used the assessment scoreboard adapted from the study by Jian Zhao and Leigh Tooth [8,9]. Details about the method used to assess research quality are provided in Appendix 1.

### 2.3 Selection process, data mining and extraction

All articles searched from databases were sifted through titles and abstracts independently by 3 researchers. Selected studies would be conducted full-text screening according to sample selection criteria, suitable studies would be extracted and included in synthesis. This study performed a data selection based on the PRISMA process of the Google search and overview research reporting system. The data selection strategy was carried out according to the following steps: Step 1: Used keywords to find all articles from databases, collected them all using Zotero, which was a fully automated citation management collection software. Step 2: Duplicate articles would be excluded using the Zotero. Step 3: Filtered articles by title and abstract. Step 4: Selected accepted articles through full-text screening. Step 5: Extracted the data and evaluate the quality of the admitted articles. Step 6: Identified articles to include in qualitative and quantitative analysis. Inclusion studies were independently filtered by the investigators using the inclusion and exclusion criteria above. In addition, we evaluated the risk of bias in observational studies using the STROBE checklist and Cochrane instrument [10,11]. If the study did not provide enough data, the author would be contacted by email and the study would be disqualified if no response was received.

Regular caffeine intake refers to the habitual consumption of caffeine-containing substances, such as coffee, tea. It implies the consistent intake of caffeine over a period of time. This is a quantitative variable that reflects a person's average caffeine consumption in milligrams [12]. For studies that control caffeine intake over a defined period of time, the study is qualified to evaluate regular caffeine intake if the subject study period is 1 month or more because when measuring long-term consumption, Studies define long-term consumption as at least one month [6,13,14]. Subjective sleep quality refers to a sense of being rested and regenerated after awaking from sleep [15]. This is an aspect that depends on each person's personal feelings and opinions. To assess subjective sleep quality, questionnaires or assessments that participants self-completed are often used.

## 3 Result

**Fig. 1:**
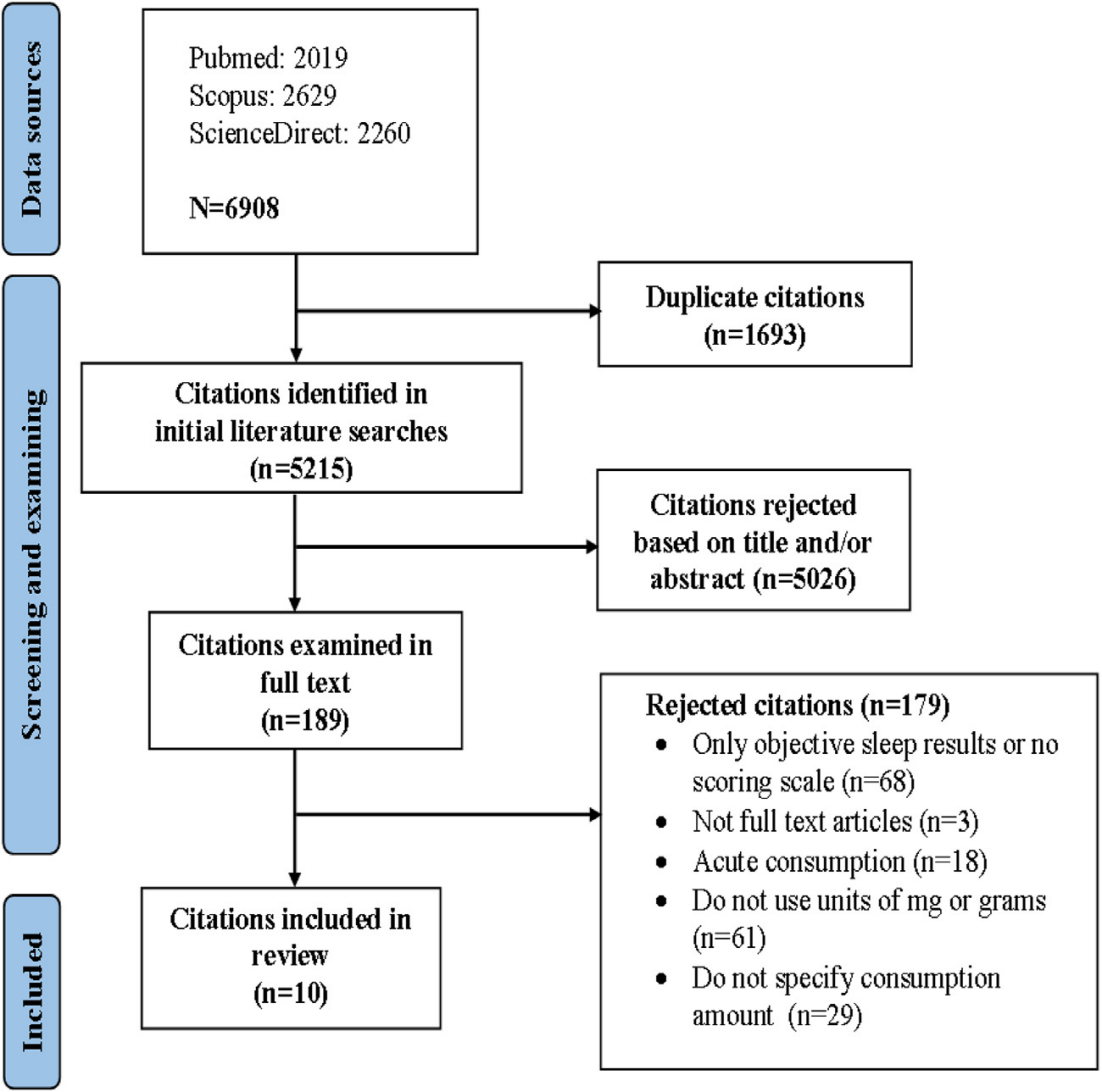
Search process.

Identified 6908 studies, of which Pubmed (2019 articles), Scopus (2629 articles), ScienceDirect (2260 articles). After removing duplicates, we continued to evaluate the titles and abstracts of 5215 studies; The remaining 189 articles were screened for full text. After screening, 179 studies were excluded. Finally, 10 studies remained were included in the literature review. The screening process was detailed in Fig. 1. Sample characteristics of the collected studies were presented in Table 1. In the study of Venkataraghavan Ramamoorthy, Emily J. Watson, Aina Riera-Sampol, Oscar H. Del Brutto, coffee was the largest source of caffeine in the study. Up to 8 out of 10 studies (80 %) published between 2016 and present, the most recent in 2022, were conducted in Spain. The absolute majority was cross-sectional study (90 %). The most commonly used scale to assess subjective sleep was PSQI. There were 3 high-quality studies (30 %). Information about quality assessment was presented in Appendix 1.

**Table 1:** Characteristics of included studies.

Main author – Year of Publication	Country	Study design	Sample size	Age	Male (%)	Average caffeine intake	Sleep quality	Quality of study
R. CURLESS – 1993	United Kingdom	Cross-section	234		49,1	360 mg/day (Community), 240 mg/ngày (Hospital)	Average score is 6, in the hospital there are 15 poor sleepers, 38 normal sleepers	Medium
H.Michael Dreher – 2003	United States of America	Randomized controlled trial	88	41,28 ± 7,87	81	Initially 476mg/day	Initially 11,06	Medium
Oscar H. Del Brutto – 2016	Ecuador	Cross-section	716	61±13	43	Unknown	4,5 ± 2,2 (203 poor sleepers)	High
Osman Mermi – 2016	Turkey	Cross-section	551	36,3 ± 12,8 (chứng), 35,5 ± 13,3 (bệnh)	43 (chứng), 37 (bệnh)	208±123 mg/day (control group), 179±146 mg/day (experimental group)	7,40±4,63 (control group), 4,51±3,13 (experimental group)	Medium
Emily J. Watson – 2016	Australia	Cross-section	80	38,9 ± 19,3	32,5	165,1 ± 105,3 mg/day	5,3 ± 2,5 (45 poor sleepers)	Medium
Venkataraghavan Ramamoorthy – 2017	United States of America	Cross-section	130	47,89±6,37	60,8	337,63 ± 304,97 mg/day	75,82 ± 47,82	Medium
Mirjam L. Kerpershoek – 2017	Netherlands	Cross-section	880	21,3 ± 3,1	25,11	624 ± 750 mg/week	5,4 ± 2,6 (39,4 % is poor sleepers)	High
Ae Kyung Chang – 2021	Korea	Cross-section	288	20–29 (200 −69,4 %); 30–40 (88 – 30,6 %)	32,3	Unknown	59,34±16,29	Medium
Liv C. Henrich – 2021	Netherlands	Cross-section	1114	21,1 ± 2,9	22,2	551±705 mg/week	5,6 (±2,8) (42,8 % is poor sleepers)	Medium
Aina Riera-Sampol-2022	Spain	Cross-section	886	20,6 ± 2,1	31	155,4 ± 173,5 mg/day	14,3 ± 3,8	High

No studies of adolescents under 18 years of age related to this topic were found. Only 2 studies had a larger number of men than women. Because exposure was characterized by long-term caffeine intake and subjective sleep quality outcomes, studies were at risk of recall bias, since the instruments were self-reported. Present studies were divided into groups based on students, adults, and other subjects.

### 3.1 Students

College students reported using caffeine-containing products to improve mood and performance or to stay awake [16]. In general, the studies found on student subjects all had large sample sizes and controlled for confounding factors, so there were 2 high-quality studies on this subject. A cross-sectional study in Netherlands, including 880 students. The study had a low selection bias because the sample loss is small, and it was not advertised through the social media accounts of a fixed number of students, so there was no tendency for those students to choose. They found that 1-week mean caffeine intake was not associated with sleep quality, after adjusting for alcohol consumption (β=−0.077, *p* = 0.071). This was the only study among the studies found that considered the time of caffeine consumption, from 6pm to 2pm. Students who avoided caffeine in the evening were found to have better sleep quality compared to those who consumed caffeine throughout the day. The study looked at multiple sources of caffeine, including the time of consumption, also controlled confounding factors (age, sex, hemp drug use, alcohol consumption). But they did not ask about the type of coffee the participants drank, since each coffee had a different caffeine content, the participants could also work part-time related to seasonal or night shifts. However, because their survey asked on a “normal” day, it avoided that participants said the amount consumed on a working day [6]. Cross-sectional study by Liv C. Henrich on other Dutch university students, with a larger sample size of 1114 students. Caffeine intake was not associated with sleep quality (*B* = 5.21E-5, *p* = 0.66). In this study, they used multivariate regression analysis including continuous variables such as caffeine consumption, categorical variables such as smoking and drug use, age and sex to control for confounding factors. The limitation of the study was that there are more women than men as well as because the recruitment process was through the social networks of sampled students, there were a high possibility of selection bias. One explanation for this result was that the amount of caffeine in the sample size was relatively low and participants in this study may have adjusted their caffeine intake based on their caffeine intake according to caffeine sensitivity [13]. Of the student studies, only one study by Aina Riera-Sampol showed an association, 886 students were sufficiently representative of students from the University of Balearic Islands, Spain. Surveys were distributed among university students through notifications on the online learning platform, 'Moodle', which limited selection bias due to volunteer accounts, broad selection criteria, sample loss was less due to not completing the questionnaire. Daily caffeine intake actually worsened sleep quality (*B* = 0.004; β=0.194; 95 %CI: 0.003–0.006; *p* < 0.001). The limitation of the study was that it was conducted in the context of limited circumstances due to COVID-19, such as limiting the opening hours of cafes, bars or restaurants, which might affect participants' responses [16].

### 3.2 Adults

All studies in adult subjects, which were of medium quality, were not very large in sample size. A study conducted in Australia, investigating the relationship between habitual caffeine consumption and sleep, including 80 people. Caffeine intake was associated with time in bed and not with subjective sleep quality. There might be a selection bias due to a large sample loss, the exclusion criteria for those who do not use caffeine daily, are not good at English may make a difference to the results. The reason why sleep quality was not related to caffeine consumption, other than those found in laboratory studies, was thought to be related to caffeine intake, as laboratory studies tend to provide amount of caffeine that is higher than the usual amount. Furthermore, because the timing of caffeine consumption was not recorded in this study, it is possible that they consumed it earlier in the day and thus had less of an effect on sleep. In addition, another limitation in this study was that the sample size of the study may not be large enough as representative of those in the caffeine-consuming group as the shift workers [12]. Ae Kyung Chang's study, conducted in Korea with a sample size of 288, the time and process of recruiting participants are not clearly described, so the risk of selection bias has not been identified. The study's exclusion criteria were that if participants were diagnosed with depression or sleep disturbance in the past 6 months and were taking medication, it is not known whether the diagnosis was consistent. For diagnostic purposes, excluding these subjects may differ in the results because people may have sleep disturbances due to caffeine consumption, so considering the relationship between caffeine consumption and sleep quality would have risk of selection bias. The study did not evaluate the association between caffeine consumption and sleep quality, but evaluated groups based on caffeine consumption; there was a difference in sleep quality between subgroups with *p* = 0.019 [17]. Research by R.Curless carried out in the elderly on 181 people in the community group and 53 patients in the hospital. The study did not mention controlling for confounding factors. There was a small negative correlation between sleep quality scores and coffee consumption (*r*=−0.2, *p* < 0.01), but not with caffeine intake. Since community enrollment comes from the age/gender register of general practitioners, it is possible that those who do not participate or are not included in the handbook, were different from the participating group, there was risk of selection bias [18].

### 3.3 Other subjects

There were a total of 4 studies that were classified as other cases because the characteristics of the sample targeted by the researcher were different from the general population in terms of pathology or culture and lifestyle.

Sleep quality in people with human immunodeficiency virus (HIV) is often worse than in the general population [19]. H. Michael Dreher's clinical trial study in the US in subjects with HIV, sample size was 88 people, divided into two groups. The experimental group would block all sources of caffeine for 30 days, control group continued using caffeine as before. The results, after adjusting for the impact of HIV-related health status, showed a 35 % improvement in sleep in the experimental group (from 11.31(SD=3.51)) to 7. 4 (SD=3.30)) (*p* < 0.001). In the control group, although there was an improvement of 7 %, applying ANCOVA analysis showed no difference before and after the time of treatment (*p* = 0,293). The study evaluated confounding factors, only 8.2 % of the samples had changes in anti-HIV drugs; therefore, it is not expected that drug changes would unduly affect the measurement of sleep dependent variables. The loss of samples during enrollment, the inclusion criteria of having a PSQI score >5 and those in the initial selection who did not agree to participate in caffeine abstinence if assigned to the experimental group could be significantly different, also leads to the risk of selection bias. In the blinding section, they had the fundamental limitation that there was no effective blinding protocol in the intervention and control groups. In addition, despite the improvement in sleep quality, when measuring the effects of caffeine reduction, it is difficult to control for the control effect if the person involved in identifying reasons for reducing caffeine consumption. However, controlling total blindness in the long run would be difficult to implement due to ethical issues [20]. Another study also included HIV-positive subjects, sampling from a Miami HIV cohort study, with a sample size of 130 people. Mean caffeine was 337.63 ± 304.97 mg/day, with no association between caffeine consumption and sleep quality scores (*p* = 0.586). Limitations are convenience sampling from another cohort study and exclusion of subjects with other comorbidities, leading to selection bias, making their study less representative of the general population. The survey instruments used in the study did not measure outcomes within the same time frame because PIRS measured insomnia levels during the past week and only measured caffeine consumption during the past 24 h. Finally, they had very few subjects who abstained from caffeine and they did not have an adequately sized non-caffeine-consuming comparison group to eliminate the effects of confounding factors influencing the relationship in this study [19].

The study by Oscar H. Del Brutto, carried out on people in rural coastal Ecuador, included 716 residents all ≥ 40 years old in the village of Atahualpa as a representative. The study did not clearly mention the time, the process of recruiting participants. The study had low selection bias due to small sample loss, sampling based on the total population in the village. They found no association between caffeine consumption and PSQI (*p* = 0.834, 95 %CI: (−0.284–0.338) and poor sleep (*p* = 0.403). For adults in rural Ecuador, caffeine intake had no effect on sleep quality. The drawback was that they did not investigate the variation in ADORA2A, which could explain the "tolerance" to caffeine consumption. The absence of an association could be explained by residents here, who lead a lifestyle with relatively small amounts of caffeine, which in turn would result in little apparent effect on sleep quality. Evaluating the association of caffeine and sleep in a large population was complicated, as there were people who think they were sensitive to coffee would not take caffeine in the evening [14].

The study was conducted in Türkiye, sampling included 401 patients with mental disorders and 150 controls. In the group of patients, caffeine consumption was associated with PSQI score (*r* = 0.158, *p* = 0.002). Their study showed that the negative effects of caffeine on mood disorders were greater in patients with mood disorders than in healthy people, and caffeine intake adversely affected sleep. Although the study had a relatively large sample size, there were still some limitations such as the heterogeneity in the diagnosis of the disorder, leading to diagnostic bias and confounding factors affecting caffeine consumption that were not evaluated [21].

## 4 Discussion

The majority of caffeine sources in the studies were from coffee, reflecting its global popularity as the second most consumed beverage after water [22]. Most studies investigating long-term caffeine intake and subjective sleep quality have been conducted in the past 7 years, focusing mainly on developed countries where caffeine consumption is culturally prevalent. However, the overall quality of evidence was moderate, largely due to limited control of confounding factors, non-representative sampling, and high attrition rates. Among the studies, four found that caffeine consumption reduced sleep quality, while six reported no significant association. Thus, current evidence is insufficient to establish a clear relationship between regular caffeine consumption and subjective sleep quality. Factors influencing these findings include how participants remember and report their caffeine intake, genetic differences (like the ADORA2A gene affecting caffeine sensitivity), and how individuals adapt to consuming caffeine over time. Additionally, participants may have adjusted their caffeine habits to minimize sleep disruptions, such as avoiding caffeine close to bedtime. This review highlights gaps in research, including the need for studies on diverse populations, such as children and infants, who may experience different caffeine metabolism. It also underscores the importance of examining the timing and form of caffeine intake, as some evidence suggests that coffee-specific compounds may affect sleep differently from caffeine alone. Limitations of this review include the exclusion of non-English studies and insufficient data for meta-analysis. Future studies should use consistent units for measuring caffeine intake and sleep quality to ensure reliable comparisons. Investigating the role of genetics and other potential confounders could also advance understanding of the caffeine-sleep relationship.

## 5 Conclusion

From reviewing 10 studies from 6908 articles across 3 databases, this review provided valuable insights into caffeine consumption and its relationship with sleep quality. The average daily caffeine intake ranged from 78.71 to 476 mg, and the prevalence of poor sleep quality ranged from 28 % to 56.25 %. However, the correlation between habitual caffeine intake and subjective sleep quality remains uncertain. To address the limitations observed, future studies should focus on the following actionable directions: Develop and adopt specific, validated scales or tools for assessing subjective sleep quality, such as the Pittsburgh Sleep Quality Index. Establish standardized units for caffeine intake measurement, including accounting for caffeine content variations in different food and beverage products. Conduct longitudinal studies incorporating both objective (e.g., actigraphy, polysomnography) and subjective measures of sleep quality to address recall and selection bias. Include representative samples from diverse populations, particularly children, adolescents, and individuals from developing countries, to ensure findings are globally applicable. Investigate genetic polymorphisms affecting caffeine metabolism (e.g., CYP1A2 gene) and their impact on individual sensitivity to caffeine. Explore cultural influences on caffeine consumption patterns and their relationship with sleep. Assess the impact of caffeine consumption timing throughout the day to better understand its role in sleep disturbances. By addressing these areas, future research can provide more definitive conclusions and improve the understanding of how caffeine consumption affects subjective sleep quality.

## 6 Glossary

1. **Caffeine**: A stimulant compound found in coffee, tea, and other beverages, known for its ability to enhance alertness and reduce drowsiness by antagonizing adenosine receptors in the brain.
2. **Subjective Sleep Quality**: An individual's perception of their sleep satisfaction, including feelings of restfulness and regeneration upon waking, often assessed through self-reported questionnaires.
3. **Pittsburgh Sleep Quality Index (PSQI)**: A standardized tool used to measure subjective sleep quality over a one-month interval, often applied in sleep-related research.
4. **Regular Caffeine Consumption**: The habitual intake of caffeine-containing products over an extended period, typically quantified in milligrams per day.
5. **Cross-Sectional Study**: A type of observational research design that analyzes data from a population at a specific point in time to examine correlations between variables.
6. **PRISMA (Preferred Reporting Items for Systematic Reviews and Meta-Analyses)**: A set of guidelines used to ensure transparency and thoroughness in systematic review processes, including study selection and data extraction.
7. **Recall Bias**: A type of error caused by differences in the accuracy or completeness of participant recollections regarding past events or experiences.
8. **ADORA2A Gene**: A gene encoding the adenosine A2A receptor, associated with individual differences in caffeine metabolism and sensitivity.
9. **Confounding Factors**: Variables that can influence both the independent and dependent variables in a study, potentially leading to biased or spurious results if not controlled for.
10. **Systematic Review**: A research methodology that synthesizes findings from multiple studies on a specific topic using a structured and reproducible approach.
11. **Randomized Controlled Trial (RCT)**: An experimental study design that randomly assigns participants to intervention or control groups to evaluate causal relationships.
12. **Sleep Hygiene**: Practices and environmental factors conducive to achieving quality sleep, such as consistent sleep schedules and avoiding caffeine before bedtime.

## Ethics approval

This systematic review was not subject to ethical approval as it did not involve any human participants.

## Funding

Not applicable.

## CRediT authorship contribution statement

**Duc Minh Phan:** Writing – review & editing, Writing – original draft, Visualization, Validation, Supervision, Software, Resources, Project administration, Methodology, Investigation, Funding acquisition, Formal analysis, Data curation, Conceptualization. **My Yen Lam:** Supervision, Methodology, Formal analysis. **Minh Nguyet Trang:** Validation, Supervision, Investigation.

## Declaration of competing interest

All authors declare no conflicts of interest in relation to this manuscript. **Duc Minh Phan:** I declare no conflicts of interest to disclose. I have no financial interest in any research or studies related to the effects of caffeine on sleep. I have not received any funding for my research from any company or organization that manufactures or sells caffeine products. **My Yen Lam:** I declare no conflicts of interest to disclose. I have no affiliations or financial involvement with any organization or entity that could be perceived to have a conflict of interest in this research. **Minh Nguyet Trang:** I declare no conflicts of interest to disclose. I have no financial or personal relationships with organizations or companies that could influence the research presented in this manuscript.

## Appendix Supplementary materials

Image, application 1

Image, application 2
