# Supplementary material for: Regular caffeine consumption & subjective sleep quality: A systematic review
Source: JAR Life. 2025 Feb 8;14:100005. doi: 10.1016/j.jarlif.2025.100005 (PMC12717775; doi:10.1016/j.jarlif.2025.100005)
Supplement: Supplementary file 1 [file mmc1.docx]

| **Number** | **Study** | **I** | **II** | **III** | **IV** | **V** | **VI** | **VII** | **VIII** | **IX** | **Total**  **score** | **Quality** |
| --- | --- | --- | --- | --- | --- | --- | --- | --- | --- | --- | --- | --- |
| 1 | Caffeine and Insomnia in People Living With HIV From the Miami Adult Studies on HIV (MASH) Cohort | 0 | 2 | 1 | 2 | 0 | 2 | 2 | 1 | 2 | **12** | Medium |
| 2 | Caffeine Consumption and Sleep Quality in Úcn Adults | 0 | 2 | 0 | 2 | 0 | 2 | 2 | 1 | 2 | **11** | Medium |
| 3 | Caffeine Intake among Undergraduate Students: Sex Differences, Sources, Motivations, and Associations with Smoking Status and Self-Reported Sleep Quality | 0 | 2 | 2 | 2 | 2 | 2 | 2 | 2 | 2 | **16** | High |
| 4 | Caffeine intake has no effect on sleep quality in community dwellers living in a rural Ecuadorian village (The Atahualpa Project) | 0 | 2 | 2 | 2 | 2 | 2 | 2 | 2 | 2 | **16** | High |
| 5 | Factors Affecting the Quality of Sleep in Young Adults | 0 | 2 | 1 | 2 | 0 | 2 | 2 | 2 | 2 | **13** | Medium |
| 6 | Evening use of caffeine moderates the relationship between caffeine consumption and subjective sleep quality in students | 0 | 2 | 2 | 2 | 2 | 2 | 2 | 2 | 2 | **16** | High |
| 7 | Is caffeine a factor in subjective insomnia of elderly people? | 0 | 2 | 1 | 2 | 0 | 2 | 2 | 1 | 2 | **12** | Medium |
| 8 | Sleep quality in students: Associations with psychological and lifestyle factors | 0 | 2 | 2 | 2 | 0 | 2 | 2 | 2 | 2 | **14** | Medium |
| 9 | The effect of caffeine reduction on sleep quality and well-being in persons with HIV | 2 | 0 | 0 | 2 | 2 | 2 | 2 | 2 | 2 | **14** | Medium |
| 10 | Habitual caffeine use in psychiatric patients: Relationship with sleep quality and symptom severity | 0 | 2 | 2 | 2 | 0 | 2 | 2 | 1 | 2 | **13** | Medium |

| **I. Study design** | **Score** |
| --- | --- |
| Prospective cohort study | 2 |
| Retrospective cohort study | 1 |
| Cross-sectional study | 0 |
|  |  |
| **II. Loss to follow** | **Score** |
| <20% | 2 |
| >20% or unknown | 0 |
|  |  |
| **III. Sample** | **Score** |
| <100 | 0 |
| >100 | 1 |
| >500 | 2 |
| **IV. Description of Period of recruitment** | **Score** |
| No | 0 |
| Yes | 2 |
| **V. Participants selection** | **Score** |
| Based on general population | 2 |
| No | 0 |
|  |  |
| **VI. Exposure** | **Score** |
| Define clearly | 2 |
| No | 0 |
|  |  |
| **VII. Outcome** | **Score** |
| Define clearly | 2 |
| No | 0 |
|  |  |
| **VIII. Statistical analysis** | **Score** |
| Proper statistical analysis with confounders controlling | 2 |
| Proper statistical analysis without confounders controlling | 1 |
| No | 0 |
|  |  |
| **IX. Result report** | **Score** |
| Summarize key results according to aims of study | 2 |
| No | 0 |
| **High: >14; Medium:11-14; Low:<11** |  |
